# Lipid profiles in adolescents with and without asthma: Korea National Health and nutrition examination survey data

**DOI:** 10.1186/s12944-018-0807-4

**Published:** 2018-07-18

**Authors:** Sun-Hye Ko, Jaewook Jeong, Myong Ki Baeg, Kyung-Do Han, Hwan Soo Kim, Jong-seo Yoon, Hyun Hee Kim, Jin Tack Kim, Yoon Hong Chun

**Affiliations:** 1 Department of Internal Medicine, Haeundae Paik Hospital, Inje University College of Medicine, Busan, Republic of Korea; 2 Department of Neurology, Gangnam Severance Hospital, Yonsei University College of Medicine, Seoul, Republic of Korea; 3 Department of Internal Medicine, International St. Mary’s Hospital, Catholic Kwandong University College of Medicine, Incheon, Republic of Korea; 4 Department of Biostatistics, College of Medicine, The Catholic University of Korea, Seoul, Republic of Korea; 5 Department of Pediatrics, Bucheon St. Mary’s Hospital, College of Medicine, The Catholic University of Korea, Bucheon, Republic of Korea; 6 Department of Pediatrics, Seoul St. Mary’s Hospital, College of Medicine, The Catholic University of Korea, Seoul, Republic of Korea; 7 Department of Pediatrics, Uijeongbu St. Mary’s Hospital, College of Medicine, The Catholic University of Korea, Uijeongbu, Republic of Korea; 8 Department of Pediatrics, Incheon St. Mary’s Hospital, College of Medicine, The Catholic University of Korea, 56, Dongsu-Ro, Bupyeong-Gu, Incheon, 21431 Republic of Korea

**Keywords:** Lipids, TG/HDL-C ratio, Asthma, Adolescence, KNHANES

## Abstract

**Background:**

Metabolic syndrome and dyslipidemia contribute to the development of a pro-inflammatory state in asthma. However, studies investigating the association between asthma and dyslipidemia have reported conflicting results. This study aimed to uncover the relationship between asthma and lipid profiles in adolescents using a national health and nutrition survey.

**Methods:**

This cross-sectional study analyzed the 2010–2012 Korea National Health and Nutrition Examination Survey data and included 2841 subjects aged 11–18 years with fasting blood sample data. Serum total cholesterol (TC), triglyceride (TG), low-density lipoprotein cholesterol (LDL-C), and high-density lipoprotein cholesterol (HDL-C) levels were analyzed. We compared asthma prevalence between high-risk and low-risk lipid groups.

**Results:**

There were 123 adolescents with asthma and 2718 without asthma (controls). The TC/HDL-C ratio, LDL-C/HDL-C ratio, and non-HDL-C levels were significantly higher in the asthma group than in the non-asthma group (*P* < 0.05). The high-risk groups displayed significantly higher asthma prevalence with higher TC, TG, LDL-C, and non-HDL-C levels and TG/HDL-C ratio than the low-risk groups (*P* < 0.05). After adjusting for potential confounding factors, the high-risk groups were associated with asthma according to their higher TC levels (adjusted odds ratio, 1.69; 95% confidence interval, 1.012–2.822) and TG/HDL-C ratios (adjusted odds ratio, 1.665; 95% confidence interval, 1.006–2.756).

**Conclusions:**

Asthma prevalence was greater in adolescents with a high TC level and TG/HDL-C ratio. In addition to the standard lipid profile, elevated TG/HDL-C ratio can be used as a useful additional lipid measure to evaluate interactions between dyslipidemia and asthma.

## Background

Asthma is a heterogeneous condition with several phenotypes [1]. Chronic airway inflammation that is characteristic of asthma can be caused by allergies, air pollution, cigarette smoke, diesel exhaust particles, aspirin, exercise, and obesity [2]. Although the underlying mechanisms are not fully understood, asthma exhibits two different disease entities: environmental allergen-related atopic asthma and allergen not-related non-atopic asthma [1, 3]. In contrast to atopic asthma, non-atopic asthma often responds poorly to inhaled or oral corticosteroids and tends to be related to more severe disease [4]. Regarding non-atopic asthma, metabolic syndrome and dyslipidemia, which are known to contribute to a pro-inflammatory state, have attracted interest as a potential cause in recent years [3, 5–7].

However, several studies investigating the relationship between lipid profiles and asthma still have reported conflicting results. A study that explored the relationship between metabolic syndrome and adult asthma showed high serum triglyceride (TG) and low serum high-density lipoprotein cholesterol (HDL-C) levels were associated with wheezing [3]. Among school children in Taiwan, asthma was found to be associated with higher low-density lipoprotein cholesterol (LDL-C) levels [8]. However, a large study of the US population demonstrated through a nationally representative survey that serum total cholesterol (TC) and non-high-density lipoprotein cholesterol (NHDL-C) levels were lower in patients with current asthma than without current asthma [9].

There is growing evidence of a higher prevalence of non-atopic asthma than of atopic asthma in children and adolescents [1, 10]. Adolescents have fewer dyslipidemia-associated comorbidities that are common in adults, such as hypertension, diabetes, and smoking [11]. Therefore, it is reasonable to examine the potential effects of dyslipidemia on asthma pathogenesis in a large representative sample of adolescents. South Korea experienced an increase of adolescent asthma and metabolic comorbidities in the past decade [12, 13]. To determine the association between dyslipidemia and asthma in adolescents, we assessed Korea National Health and Nutrition Examination Survey (KNHANES) data. KNHANES is a nationally representative cross-sectional survey conducted to estimate the health and nutritional status of the Korean population.

## Methods

### Data source and subjects

This cross-sectional study was based on the fifth KNHANES (KNHANES V) conducted from 2010 to 2012. KNHANES V consisted of a health interview, health examination survey, and nutrition survey [14]. Additional details regarding the study design and methods are provided elsewhere [15]. This study used a rolling sample design with stratified multistage cluster-probability sampling. A total of 31,596 individuals were sampled for KNHANES V, and 25,533 participated, resulting in a response rate of 80.8%. Among them, 3443 subjects aged 11–18 years were selected. The exclusion criteria were insufficient fasting time (*n* = 350) and missing data for variables included in the analysis (*n* = 252). Finally, 2841 subjects were analyzed, and 123 were diagnosed with asthma by a physician. The institutional review board of the Korea Centers for Disease Control and Prevention approved the protocol and all participants signed informed consent forms.

### Measurements

Questionnaires were administered to gather data on demographic characteristics, smoking status, alcohol consumption, daily exercise level, residential status (urban or rural), parental income, sleep duration, and daily nutritional intake. Based on self-reported smoking behavior, the subjects were categorized as current smokers or nonsmokers. Based on alcohol consumption, they were classified as non-drinkers, mild-to-moderate drinkers (1.0–30.0 g alcohol/day), or heavy drinkers (≥30.0 g alcohol/day) [14]. Based on their responses to a modified version of the International Physical Activity Questionnaire, the subjects were classified as regular or non-regular exercisers. Parental income was inflation-adjusted and measured as quartiles to classify subjects into the highest, middle-high, middle-low, and lowest quartiles [14]. Sleep duration was determined according to the answer to the question, “How much time do you usually sleep in a day?” Daily energy intake and fat intake were assessed using a 24-h dietary-recall method.

Anthropometric measurements were performed by specially trained examiners. Height and weight were measured, and body mass index (BMI) was calculated as weight in kilograms divided by the square of height in meters. Waist circumference was measured to the nearest 0.1 cm in a horizontal plane at the midpoint between the iliac crest and the costal margin. Blood pressure was measured in subjects seated for at least 5 min, using a mercury sphygmomanometer on the right arm (Baumanometer; Baum, Copiague, NY, USA).

Blood samples were collected from the subjects after fasting for at least 8 h, were immediately refrigerated, and were transported in low-temperature storage at the Central Testing Institute in Seoul, Korea. Serum fasting blood sugar, HDL-C, LDL-C, TC, and TG concentrations were measured by enzymatic methods (Hitachi Automatic Analyzer 7600; Hitachi, Tokyo, Japan). NHDL-C levels were derived by subtracting the HDL-C concentration from the TC concentration. The TC/HDL-C, LDL-C/HDL-C, and TG/HDL-C ratios were calculated by dividing the TC, LDL-C, and TG levels by the HDL-C levels, respectively.

### Definitions

Asthma was defined by self-reporting of a diagnosis by the physician. We used the following survey question in this study: “Have you ever had asthma diagnosed by a physician in the past?” The subjects were stratified into quartiles for each of the eight lipid profiles (TC, TG, LDL-C, HDL-C, TC/HDL-C, TG/HDL-C, LDL-C/HDL-C, and NHDL-C). For all lipid profiles except HDL-C levels, we defined groups with the highest quartile (Q4) as “high-risk groups.” For HDL-C levels, we defined groups with the lowest quartile (Q1) as “high-risk groups.” The rest were defined as the “low-risk groups.” Additionally, we divided the participants into four groups to compare asthma prevalence according to the number of high-risk groups for their TC, TG, LDL-C, and HDL-C levels. The four groups were as follows: having no high-risk groups, having one high-risk group, having two high-risk groups, and having three or four high-risk groups. Asthma prevalence was calculated by dividing the number of subjects with asthma by the number of high-risk groups.

### Statistical analysis

We used the KNHANES stratification variables and sampling weights identified by the Korea Centers for Disease Control and Prevention, which were based on the sample design for each survey year [14]. Data were expressed as means ± standard error for continuous variables and as percentages for categorical variables, unless otherwise stated. Variables with skewed distributions were analyzed after logarithmic transformation.

Multivariate logistic regression was performed to analyze the association between serum lipid concentrations and asthma prevalence. The risk of asthma in the high-risk groups for each lipid was compared with those in the low-risk groups (high-risk groups: Q4 of TC, LDL-C, TG, TC/HDL-C, LDL-C/HDL-C, TG/HDL-C, and NHDL-C, and Q1 of HDL-C; low-risk groups: the rest of the quartiles). Three models were constructed for each lipid: Model 1 included adjustments for age and sex and model 2 additional adjustments for place of residence, regular exercise, lowest income, and sleep duration; model 3 was the same as model 2 with the addition of adjustments for energy intake and fat intake. Results were presented as odds ratios (OR) and 95% confidence intervals (CI). Data analysis was performed using SAS for Windows (version 9.20, SAS Institute, Cary, NC, USA), and *P* < 0.05 indicated statistical significance.

## Results

### Characteristics of the participants

The demographic characteristics of the study population are summarized in Table 1. The study population comprised 2841 participants (1511 male and 1330 female) and the mean age was 15.1 years. Among them, 123 were diagnosed with asthma (asthma group) and 2718 were not (non-asthma group). Compared with the non-asthma group, the asthma group had a higher tendency to be residing in an urban area, had a higher energy and fat intake, and were more likely to be males (*P* = 0.0313, 0.0195, 0.0128 and 0.0049, respectively). Moreover, the asthma group had higher TC/HDL-C and LDL-C/HDL-C ratios and NHDL-C level than the non-asthma group (*P* = 0.014, 0.0277, and 0.0499, respectively). However, there was no significant difference in TC, TG, HDL-C, and LDL-C levels between both groups.

**Table 1:** Clinical characteristics of the participants

	Non-asthma group	Asthma group	*P* value
	(*n* = 2718)^a^	(*n* = 123)^a^	
Age (years)	15.0 ± 0.0	15.1 ± 0.2	0.8436
Male (%)^b^	53.3 (1.1)	67.3 (4.5)	0.0049
BMI (kg/m^2^)	21.0 ± 0.1	21.7 ± 0.5	0.1448
Waist circumference (cm)	70.8 ± 0.2	73.3 ± 1.3	0.0501
Systolic BP (mmHg)	107.2 ± 0.3	108.9 ± 1.2	0.1648
Diastolic BP (mmHg)	67.9 ± 0.2	69.7 ± 1.0	0.0823
Smoker, ever (%)	18.6 (0.9)	15.0 (4.4)	0.4536
Drinking, mild to moderate (%)	28.1 (1.1)	20.3 (4.3)	0.1077
Regular exercise (%)	29.8 (1.0)	32.8 (5.4)	0.5728
Income, lowest quartile (%)	15.0 (1.1)	9.7 (3.4)	0.1781
Rural area (%)^b^	17.4 (2.0)	9.2 (3.0)	0.0313
Sleep duration (hours)	7.2 ± 0.0	7.1 ± 0.2	0.6538
Energy intake (kcal)^b^	2104.6 ± 22.4	2306.0 ± 83.5	0.0195
Fat intake (%)^b^	22.5 ± 0.2	20.7 ± 0.7	0.0128
TC (mg/dL)	155.5 ± 0.7	160.5 ± 3.6	0.1679
TG (mg/dL)^c^	74.1 (72.3–76.1)	83.5 (73.7–94.5)	0.0656
HDL-C (mg/dL)	53.1 ± 0.3	51.1 ± 1.0	0.0529
LDL-C (mg/dL)	85.6 ± 0.6	89.9 ± 2.9	0.1352
NHDL-C (mg/dL)^b^	102.4 ± 0.7	109.4 ± 3.5	0.0499
TC/HDL-C^b^	3.0 ± 0.0	3.2 ± 0.1	0.0142
LDL-C/HDL-C^b^	1.7 ± 0.0	1.8 ± 0.1	0.0277
White blood cells (1000/uL)^c^	6 (6–6.1)	6.2 (5.8–6.6)	0.5115
Ferritin (ng/mL)^c^	32.4 (31.1–33.7)	36.3 (31.6–41.7)	0.1183

*BMI* body mass index; *BP* blood pressure; *TC* total cholesterol; *TG* triglyceride; *HDL-C* high-density lipoprotein cholesterol; *LDL-C* low-density lipoprotein cholesterol; *NHDL-C* non-high-density lipoprotein cholesterol

^a^Data are presented as means ± standard error (SE) or percentages and SE

^b^*P* < 0.05

^c^Geometric mean (95% confidence interval)

### Asthma prevalence according to lipid groups

**Fig. 1:**
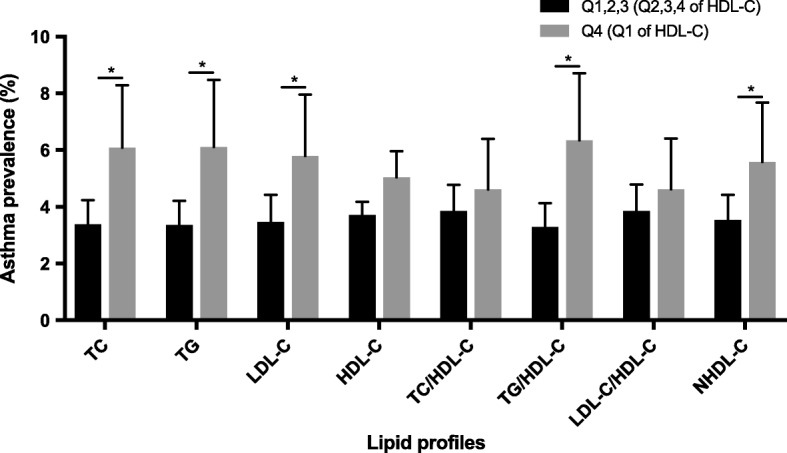
Asthma prevalence in low-risk vs. high-risk groups. The high-risk groups consisted of the highest quartile (Q4) of TC, TG, LDL-C, TC/HDL-C, TG/HDL-C, LDL-C/HDL-C, and NHDL-C levels and ratios and the lowest quartile (Q1) of HDL-C levels. The low-risk groups consisted of the rest of the three quartiles. *P < 0.05. TC, total cholesterol; TG, triglyceride; HDL-C, high-density lipoprotein cholesterol; LDL-C, low-density lipoprotein cholesterol; NHDL-C, non-high-density lipoprotein cholesterol

**Fig. 2:**
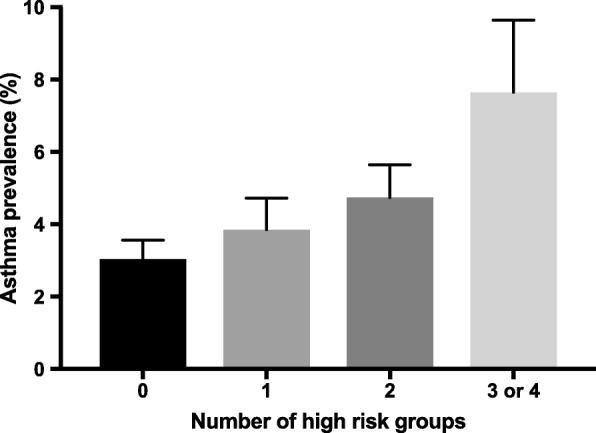
Asthma prevalence according to the number of high-risk groups. The height of each bar represents asthma prevalence for no high-risk groups (0), one high-risk group (1), two high-risk groups (2), and three or four high-risk groups (3 or 4). The four high-risk group consist of the highest quartile (Q4) of total cholesterol, triglyceride, low-density lipoprotein cholesterol, and the lowest quartile (Q1) of high-density lipoprotein cholesterol. Asthma prevalence increased with increasing number of high-risk groups (all *P* for trend = 0.005)

Asthma prevalence in the high-risk groups was compared with that in the low-risk groups (Fig. 1). Asthma was more prevalent in the high-risk groups for serum TC, TG, LDL-C, TG/HDL-C, and NHDL-C (*P* = 0.006, 0.010, 0.021, 0.004, and 0.041, respectively). Though not statistically significant, asthma prevalence tended to be higher in the high-risk group for HDL-C (5.038 vs. 3.712, *P* = 0.160). We assessed asthma prevalence according to the number of high-risk groups for the TC, TG, LDL-C, and HDL-C levels (Fig. 2). Regarding participants who did not meet the criteria for any of the four high-risk groups, the asthma prevalence was 3.031. For participants who met the criteria for one high-risk group, two high-risk groups, and three or four high-risk groups, the asthma prevalence was 3.852, 4.741, and 7.644, respectively (*P* for trend = 0.005). Thus, asthma prevalence linearly increased with increasing number of high-risk groups.

### Association between asthma and lipids

Table 2 demonstrates the risk of asthma in the high-risk group for each lipid compared with the low-risk group. On age- and sex-adjusted logistic regression analysis (model 1), the high-risk groups for TC, TG, LDL-C, TG/HDL-C, and NHDL-C had a higher risk of asthma than the low-risk groups. These associations remained significant after further adjustment for place of residence, BMI, regular physical activity, parental income, and sleep duration (model 2). After adjustment for daily energy intake and fat intake (model 3), the ORs for having asthma were 1.69 (95% CI, 1.012–2.822) in the high-risk TC group and 1.665 (95% CI, 1.006–2.756) in the high-risk TG/HDL-C group, compared with the low risk group. In model 3, the association between asthma and the high-risk TG, LDL-C and NHDL-C groups was no longer significant.

**Table 2:** Adjusted odds ratios for asthma prevalence according to the quartile groups of lipids

	Model 1	Model 2	Model 3
TC (Q4 vs. Q1, 2, 3)	1.859 (1.184, 2.918)^a^	1.863 (1.169, 2.97)^a^	1.69 (1.012, 2.822)^a^
TG (Q4 vs. Q1, 2, 3)	1.89 (1.165, 3.068)^a^	1.66 (1.057, 2.607)^a^	1.444 (0.876, 2.378)
LDL-C (Q4 vs. Q1, 2, 3)	1.715 (1.079, 2.725)^a^	1.698 (1.055, 2.732)^a^	1.585 (0.946, 2.655)
HDL-C (Q1 vs. Q2, 3, 4)	1.374 (0.879, 2.149)	1.364 (0.875, 2.125)	1.335 (0.817, 2.18)
TC/HDL-C (Q4 vs. Q1, 2, 3)	1.214 (0.776, 1.901)	1.152 (0.731, 1.816)	0.947 (0.568, 1.578)
TG/HDL-C (Q4 vs. Q1, 2, 3)	2.024 (1.253, 3.269)^a^	1.809 (1.143, 2.862)^a^	1.665 (1.006, 2.756)^a^
LDL-C/HDL-C (Q4 vs. Q1, 2, 3)	1.21 (0.771, 1.9)	1.137 (0.718, 1.8)	1.01 (0.61, 1.67)
NHDL-C (Q4 vs. Q1, 2, 3)	1.627 (1.025, 2.584)^a^	1.617 (1.002, 2.61)^a^	1.437 (0.848, 2.438)

Model 1: adjusted for age and sex. Model 2: adjusted for age, sex, place of residence, body mass index, regular physical activity, lowest income, and sleep duration. Model 3: adjusted for age, sex, place of residence, body mass index, regular physical activity, lowest income, sleep duration, energy intake, and fat intake

TC, total cholesterol; TG, triglyceride; HDL-C, high-density lipoprotein cholesterol; LDL-C, low-density lipoprotein cholesterol; NHDL-C, non-high-density lipoprotein cholesterol

^a^*P* < 0.05

## Discussion

This study revealed positive relationships between dyslipidemia and asthma in a large, representative sample of adolescents. Higher serum TC, TG, LDL-C, NHDL-C levels and TG/HDL-C ratio were associated with higher prevalence of asthma. Particularly, TC level and TG/HDL-C ratio maintained positive correlations with asthma even after adjusting for potential, confounding factors on multivariate analysis.

Combined dyslipidemia is the predominant pattern of lipid abnormality in adolescents [11]. Besides traditional lipid profile measures, elevated NHDL-C and TG/HDL-C ratios are considered as important, additional lipid measures for the diagnosis of combined dyslipidemia in childhood [11]. An elevated TG/HDL-C ratio is known to be correlated with insulin resistance, non-alcoholic fatty-liver disease and atherosclerosis in children [16]. However, there are no previous reports that show the relationship between TG/HDL-C and risk of asthma.

TC and HDL-C data are reliable lipid profile measures in pediatric patients, who often report inaccurate fasting times for blood tests [11]. In this study, the high risk group of TC showed high prevalence of asthma. The findings of several studies are consistent with our results. Hypercholesterolemia was found to increase the probability of asthma in both adolescents and adults [6, 17]. Additionally, a Taiwan study reported a positive relationship between TC level and asthma development only in boys [8]. Although we observed more males in the asthma group compared to the non-asthma group, the relationship between TC level and asthma prevalence remained after age- and sex-adjusted logistic regression analysis.

Currently, the mechanisms behind the association between dyslipidemia and asthma or other allergic diseases remain unclear; however, some potential mechanisms exist. One mechanism could be the inflammatory link between asthma and dyslipidemia. Asthma is characterized by chronic airway inflammation. Meanwhile, hypercholesterolemia plays a pro-inflammatory role, inducing the release of inflammatory cytokines and upregulating cellular adhesion molecules in the endothelium [18, 19]. Furthermore, dyslipidemia may also potentiate eosinophilic inflammation that is implicated in other conditions of asthma pathophysiology, such as mucus hypersecretion, bronchial hyperresponsiveness, and subepithelial fibrosis [6, 20–22].

Another mechanism that explains the association between dyslipidemia and asthma may be cholesterol trafficking and a switch to innate immune response [23, 24]. Cholesterol is an essential component of lipid rafts, which are microdomains of the cell membrane that play an important role in cell signaling [23, 25]. Slight changes to cholesterol in these rafts can trigger the toll-like receptor-signaling pathway in macrophages to enhance immune reaction [23, 24]. Furthermore, a study reported that hypercholesterolemia is associated with a T- helper (Th)1/Th2 switch of the autoimmune response in cholesterol-fed apo E-knockout mice [26]. This switched immune response could explain the vulnerability of adolescents with dyslipidemia to the development of airway remodeling, which is a characteristic inflammatory change in patients with asthma.

This study has some limitations. First, because of the cross-sectional nature of the KNHANES, the association between asthma and dyslipidemia may not imply a causal relationship. Second, we could not analyze the data according to the asthma phenotype (i.e., atopic or non-atopic asthma) due to the lack of specific immunoglobulin E or skin-prick test data. Third, because the original data did not include a detailed description of medication use or pulmonary function data, we could not determine the relationship between asthma severity and dyslipidemia. Furthermore, we could not include parental asthma in logistic regression analysis because the survey did not investigate family history of asthma in adolescents. Therefore, future longitudinal studies are needed to determine the temporal relationship between dyslipidemia and asthma prevalence.

Despite these limitations, we used a highly reliable, nationally representative sample of a homogeneous society, which is a major strength of our study [27]. In addition, asthmatics showed definite positive relationships between dyslipidemia and asthma even though, in our study, they had normal BMI and waist circumference. Several other studies that reported similar results only targeted obese patients [3, 5, 7, 8]. This finding may suggest that dyslipidemia is a risk factor independent of obesity. Another strength is that the subjects were adolescents with a narrow age range and fewer comorbidities that can affect dyslipidemia status. By considering that adults are more likely to have had chronic inflammation and atherosclerotic changes, the interpretation of this study that dyslipidemia may be a risk factor for asthma becomes more reliable [28, 29].

## Conclusion

In conclusion, hyperlipidemia showed positive association with asthma prevalence in Korean adolescents. Especially, higher serum TC level and TG/HDL-C ratio are independent risk factors for asthma in this study. To our knowledge, this is the first large population-based study that reports a positive association between TG/HDL-C ratio and asthma in adolescents. In addition to standard lipid profiles, elevated TG/HDL-C ratio can be a useful, additional lipid measure to evaluate interactions between dyslipidemia and asthma.

## Abbreviations

BMI: body mass index
BP: blood pressure
CI: confidence interval
HDL-C: high-density lipoprotein cholesterol
KNHANES: Korea National Health and Nutrition Examination Survey
LDL-C: low-density lipoprotein cholesterol
LDL-C/HDL-C: low-density lipoprotein cholesterol/high-density lipoprotein cholesterol ratio
NHDL-C: non-high-density lipoprotein cholesterol
OR: odds ratio
Q: quartile
SE: standard error
TC: total cholesterol
TC/HDL-C: total cholesterol/high-density lipoprotein cholesterol ratio
TG: triglyceride
TG/HDL-C: triglyceride/high-density lipoprotein cholesterol ratio
Th: T-helper

## References

[1] Lawson JA, Chu LM, Rennie DC, Hagel L, Karunanayake CP, Pahwa P (2017). Prevalence, risk factors, and clinical outcomes of atopic and nonatopic asthma among rural children. Ann Allergy Asthma Immunol.

[2] Kim HY, DeKruyff RH, Umetsu DT (2010). The many paths to asthma: phenotype shaped by innate and adaptive immunity. Nat Immunol.

[3] Fenger RV, Gonzalez-Quintela A, Linneberg A, Husemoen LL, Thuesen BH, Aadahl M (2013). The relationship of serum triglycerides, serum HDL, and obesity to the risk of wheezing in 85,555 adults. Respir Med.

[4] de Llano LP, del Vennera M C, Álvarez FJ, Medina JF, Borderías L, Pellicer C (2013). Spanish registry.. Effects of omalizumab in non-atopic asthma: results from a Spanish multicenter registry. J Asthma.

[5] Lugogo NL, Bappanad D, Kraft M (2011). Obesity, metabolic dysregulation and oxidative stress in asthma. Biochim Biophys Acta.

[6] Ramaraju K, Krishnamurthy S, Maamidi S, Kaza AM, Balasubramaniam N (2013). Is serum cholesterol a risk factor for asthma?. Lung India.

[7] Zeki AA, Kenyon NJ, Goldkorn T (2011). Statin drugs, metabolic pathways, and asthma: a therapeutic opportunity needing further research. Drug Metab Lett.

[8] Chen YC, Tung KY, Tsai CH, Su MW, Wang PC, Chen CH (2013). Lipid profiles in children with and without asthma: interaction of asthma and obesity on hyperlipidemia. Diabetes Metab Syndr.

[9] Fessler MB, Massing MW, Spruell B, Jaramillo R, Draper DW, Madenspacher JH (2009). Novel relationship of serum cholesterol with asthma and wheeze in the United States. J Allergy Clin Immunol.

[10] Su YT, Yang YN, Li YC, Tsai CC, Chen LM, Lin YC (2016). Age-dependent distribution of the atopic phenotype and allergen sensitization among asthmatic children in southern Taiwan. Asian Pac J Allergy Immunol.

[11] Kavey RE (2015). Combined dyslipidemia in childhood. J Clin Lipidol.

[12] Lim H, Xue H, Wang Y (2014). Association between obesity and metabolic co-morbidities among children and adolescents in South Korea based on national data. BMC Public Health.

[13] Yoo B, Park Y, Park K, Kim H (2015). A 9-year trend in the prevalence of allergic disease based on national health insurance data. J Prev Med Public Health.

[14] Ministry of Health and Social Welfare, National Health and Nutrition Survey. Report on 1998 First National Health and Nutrition Survey (approval no. 11702). National Health and Nutrition Survey. Available at: http://cdc.go.kr/CDC/contents/CdcKrContentView.jsp?cid=60942&menuIds=HOME001-MNU1130-MNU1639-MNU1748-MNU1754. Accessed: 3 Jan 2014.

[15] Choi YJ, Lee MS, An SY, Kim TH, Han SJ, Kim HJ (2011). The relationship between diabetes mellitus and health-related quality of life in Korean adults: the fourth Korea National Health and nutrition examination survey (2007-2009). Diabetes Metab J.

[16] Pacifico L, Bonci E, Andreoli G, Romaggioli S, Di Miscio R, Lombardo CV (2014). Association of serum triglyceride-to-HDL cholesterol ratio with carotid artery intima-media thickness, insulin resistance and nonalcoholic fatty liver disease in children and adolescents. Nutr Metab Cardiovasc Dis.

[17] Al-Shawwa B, Al-Huniti N, Titus G, Abu-Hasan M (2006). Hypercholesterolemia is a potential risk factor for asthma. J Asthma..

[18] Baumruker T, Csonga R, Pursch E, Pfeffer A, Urtz N, Sutton S (2003). Activation of mast cells by incorporation of cholesterol into rafts. Int Immunol.

[19] Scalia R, Appel JZ, Lefer AM (1998). Leukocyte-endothelium interaction during the early stages of hypercholesterolemia in the rabbit: role of P-selectin, ICAM-1, and VCAM-1. Arterioscler Thromb Vasc Biol.

[20] Pham TH, Damera G, Newbold P, Ranade K (2016). Reductions in eosinophil biomarkers by benralizumab in patients with asthma. Respir Med.

[21] Humbles AA, Lloyd CM, McMillan SJ, Friend DS, Xanthou G, McKenna EE (2004). A critical role for eosinophils in allergic airways remodeling. Science.

[22] Kay AB, Phipps S, Robinson DS (2004). A role for eosinophils in airway remodelling in asthma. Trends Immunol.

[23] Ikonen E (2008). Cellular cholesterol trafficking and compartmentalization. Nat Rev Mol Cell Biol.

[24] Fessler MB, Parks JS (2011). Intracellular lipid flux and membrane microdomains as organizing principles in inflammatory cell signaling. J Immunol.

[25] Horejsi V, Hrdinka M (2014). Membrane microdomains in immunoreceptor signaling. FEBS Lett.

[26] Zhou X, Paulsson G, Stemme S, Hansson GK (1998). Hypercholesterolemia is associated with a T helper (Th) 1/Th2 switch of the autoimmune response in atherosclerotic apo E-knockout mice. J Clin Invest.

[27] Kim Y (2014). The Korea National Health and nutrition examination survey (KNHANES): current status and challenges. Epidemiol Health.

[28] Onufrak S, Abramson J, Vaccarino V (2007). Adult-onset asthma is associated with increased carotid atherosclerosis among women in the atherosclerosis risk in communities (ARIC) study. Atherosclerosis.

[29] Knoflach M, Kiechl S, Mayr A, Willeit J, Poewe W, Wick G (2005). Allergic rhinitis, asthma, and atherosclerosis in the Bruneck and ARMY studies. Arch Intern Med.

